# The relationship between parameters measured using intravoxel incoherent motion and dynamic contrast-enhanced MRI in patients with breast cancer undergoing neoadjuvant chemotherapy: a longitudinal cohort study

**DOI:** 10.3389/fonc.2024.1356173

**Published:** 2024-05-22

**Authors:** Zyad M. Almutlaq, Sarah E. Bacon, Daniel J. Wilson, Nisha Sharma, Tatendashe Dondo, David L. Buckley

**Affiliations:** ^1^ Biomedical Imaging, Leeds Institute of Cardiovascular and Metabolic Medicine (LICAMM), University of Leeds, Leeds, United Kingdom; ^2^ Radiological Sciences Department, College of Applied Medical Sciences, King Saud bin Abdulaziz University for Health Sciences, Riyadh, Saudi Arabia; ^3^ Department of Medical Physics & Engineering, Leeds Teaching Hospitals National Health Service (NHS) Trust, Leeds, United Kingdom; ^4^ Department of Radiology, Leeds Teaching Hospitals NHS Trust, Leeds, United Kingdom; ^5^ Clinical and Population Sciences Department, LICAMM, University of Leeds, Leeds, United Kingdom

**Keywords:** breast cancer, intravoxel incoherent motion, dynamic contrast enhanced MRI, perfusion, repeated measures, correlations

## Abstract

**Purpose:**

The primary aim of this study was to explore whether intravoxel incoherent motion (IVIM) can offer a contrast-agent-free alternative to dynamic contrast-enhanced (DCE)-MRI for measuring breast tumor perfusion. The secondary aim was to investigate the relationship between tissue diffusion measures from DWI and DCE-MRI measures of the tissue interstitial and extracellular volume fractions.

**Materials and methods:**

A total of 108 paired DWI and DCE-MRI scans were acquired at 1.5 T from 40 patients with primary breast cancer (median age: 44.5 years) before and during neoadjuvant chemotherapy (NACT). DWI parameters included apparent diffusion coefficient (ADC), tissue diffusion (D_t_), pseudo-diffusion coefficient (D_p_), perfused fraction (f), and the product f×D_p_ (microvascular blood flow). DCE-MRI parameters included blood flow (F_b_), blood volume fraction (v_b_), interstitial volume fraction (v_e_) and extracellular volume fraction (v_d_). All were extracted from three tumor regions of interest (whole-tumor, ADC cold-spot, and DCE-MRI hot-spot) at three MRI visits: pre-treatment, after one, and three cycles of NACT. Spearman’s rank correlation was used for assessing between-subject correlations (r), while repeated measures correlation was employed to assess within-subject correlations (r_rm_) across visits between DWI and DCE-MRI parameters in each region.

**Results:**

No statistically significant between-subject or within-subject correlation was found between the perfusion parameters estimated by IVIM and DCE-MRI (f versus v_b_ and f×D_p_ versus F_b_; P=0.07–0.81). Significant moderate positive between-subject and within-subject correlations were observed between ADC and v_e_ (r=0.461, r_rm_=0.597) and between D_t_ and v_e_ (r=0.405, r_rm_=0.514) as well as moderate positive within-subject correlations between ADC and v_d_ and between D_t_ and v_d_ (r_rm_=0.619 and 0.564, respectively) in the whole-tumor region.

**Conclusion:**

No correlations were observed between the perfusion parameters estimated by IVIM and DCE-MRI. This may be attributed to imprecise estimates of fxD_p_ and v_b_, or an underlying difference in what IVIM and DCE-MRI measure. Care should be taken when interpreting the IVIM parameters (f and f×D_p_) as surrogates for those measured using DCE-MRI. However, the moderate positive correlations found between ADC and D_t_ and the DCE-MRI parameters v_e_ and v_d_ confirms the expectation that as the interstitial and extracellular volume fractions increase, water diffusion increases.

## 1 Introduction

Breast cancer is one of the most prevalent cancers affecting women globally, with about 2.3 million women diagnosed with the disease and 685,000 deaths in 2020 (1). Patients with primary breast cancer are often treated with neoadjuvant chemotherapy (NACT) to downsize the tumor and increase the probability of breast-conserving surgery (2). A non-invasive imaging technique that can provide information on tumor cellularity and perfusion during treatment would be beneficial, as reduced cellularity and perfusion are promising indicators of patient response to treatment (3).

Patients with breast cancer undergoing NACT often undergo repeated dynamic contrast-enhanced (DCE) MRI scans for treatment monitoring (4). DCE-MRI is a widespread technique that can provide information on tumor perfusion and cellularity through serial MRI scans acquired before and after the injection of a gadolinium-based contrast agent (3). Furthermore, quantitative estimation of perfusion-related parameters of breast tumors, including tumor blood flow (F_b_), blood volume fraction (v_b_), along with hemodynamic and cellularity-related parameters: capillary permeability–surface area product (PS); interstitial volume fraction (v_e_), and extracellular volume fraction (v_d_; calculated from the combination of blood volume and interstitial volume fractions) can be achieved by employing a recently developed DCE-MRI technique (5). However, certain safety concerns exist regarding gadolinium administration, particularly in patients with cancer who undergo repeated contrast-enhanced scans (6). Therefore, alternative imaging techniques that can provide equivalent perfusion and cellularity-related measurements without administering a contrast agent are of interest.

Conventional diffusion-weighted imaging (DWI), which is not used generally in breast cancer imaging, can be employed in oncology treatment response monitoring through the apparent diffusion coefficient (ADC). The ADC measures the diffusivity of water molecules in the tissue and is assumed to serve as an indicator of cellular density. As such, as tumor cellularity decreases in response to treatment, the ADC value increases (7). The ADC is therefore expected to be directly proportional to the DCE-MRI measurements of the tissue’s interstitial and extracellular volume fractions. However, few studies have examine this relationship, and one study in breast tumors has challenged the expectation suggesting that the ADC is incompletely understood (8, 9). Also, blood in the microcirculation can contaminate the DWI signal decay, contributing to the ADC value (10). A technique that can potentially address the problems affecting both DCE-MRI and DWI is intravoxel incoherent motion (IVIM), an advanced form of DWI. It has been proposed that IVIM enables simultaneous assessment of tissue diffusion and perfusion by separating the effects of the microcirculation of blood in the capillary network (so-called pseudo-diffusion) from water diffusion in the rest of the tissue. This method requires DWI acquisitions with multiple b-values (low and high) and fits a bi-exponential model to the data to estimate the diffusion-related parameter D_t_ (tissue diffusion) and perfusion-related parameters, including D_p_ (the pseudo-diffusion coefficient), f (the perfused fraction), and their product f×D_p_ (microvascular blood flow) (10, 11).

In the past decade, growing interest in exploring the potential applications of IVIM in breast tumors has produced studies differentiating benign and malignant tumors (12, 13). IVIM perfusion-related parameters have also shown some promise for evaluating breast tumor response to NACT (14–16). This in turn has reopened the question of whether IVIM could be used as a contrast-agent-free alternative to DCE-MRI for measuring breast tumor perfusion. Few studies have investigated the correlations between IVIM and DCE-MRI perfusion-related parameters in breast tumors and have produced contradictory results (17–19). These studies examined correlations at a single visit; however, a correlation between perfusion parameter changes caused by treatment is meaningful and suggests that IVIM could be a contrast-agent-free surrogate to the DCE-MRI method in monitoring serial changes in tumor perfusion. Further, none of these studies provided an absolute estimation of tumor blood flow; they did not perform a direct comparison with the IVIM parameter purported to measure microvascular blood flow (f×D_p_).

The primary aim of this study was to investigate whether IVIM and DCE-MRI perfusion-related parameters correlate and whether IVIM can offer a contrast-agent-free alternative to DCE-MRI for monitoring serial changes in tumor perfusion. The DCE-MRI data were analyzed to estimate absolute tumor blood flow, blood volume fraction, capillary permeability–surface area product, interstitial volume fraction, and extracellular volume fraction (5). This study assesses both between-subject and within-subject repeated measures correlations between the perfusion parameters estimated by IVIM and DCE-MRI (specifically perfusion fraction versus blood volume fraction and microvascular blood flow versus blood flow) in a cohort of patients with breast cancer imaged before treatment and after one and three cycles of NACT. Analyzing both correlations is valuable; between-subject correlation reveals the potential for estimating DCE-MRI perfusion parameters using IVIM at a given time, whereas within-subject repeated measures correlations indicate the potential for estimating change in DCE-MRI perfusion parameters using IVIM when assessing longitudinal changes in the same patient. The secondary aim of this study was to examine the correlation between tissue diffusion measures from DWI and DCE-MRI measures of the tissue’s interstitial and extracellular volume fractions. This would improve the understanding of tissue diffusion measures and their changes in response to treatment further, which are of interest for translation into breast cancer imaging as markers of treatment response (20).

## 2 Materials and methods

### 2.1 Patients

The prospective study had local research ethics committee approval, and written informed consent was obtained from all subjects. The eligibility criteria for patient inclusion were: 1) 18 years of age and older; 2) pathological confirmation of primary invasive breast cancer through a core needle biopsy; and 3) scheduled to undergo NACT. Patients who had impaired kidney function or contraindications to MRI were considered ineligible. Recruited patients underwent a standardized NACT regimen consisting of three cycles of epirubicin (90 mg/m^2^) and cyclophosphamide (600 mg/m^2^) (one cycle every three weeks), followed by three cycles of docetaxel (100 mg/m^2^, one cycle every three weeks). Patients with tumors positive for human epidermal growth factor receptor 2 were treated with trastuzumab and/or pertuzumab alongside docetaxel.

### 2.2 Image acquisition

MRI scans were performed at baseline (pre-treatment) and after one and three (mid-treatment) cycles of NACT. All images were acquired using a 1.5-T MRI scanner (Aera; Siemens) with the patient in a head-first prone position. A dedicated 16-channel breast coil (Sentinelle; Siemens) was used to image the breasts, and a flexible array coil, placed on the patient’s back, was employed to increase the signal from the descending aorta (21). The scanning protocol included axial T_2_-weighted turbo spin-echo, axial T_1_-weighted 3D spoiled gradient echo, inversion recovery, DWI, and DCE-MRI sequences.

The axial DWI was acquired using a spectral attenuated inversion recovery fat-suppressed, 2D single-shot spin-echo echo-planar imaging sequence (repetition time/echo time: 7200/59 ms, flip angle: 90°, field of view: 340×136×169 mm, matrix size: 280×116×34, slice thickness: 4 mm, acceleration factor: 2, acquisition time: 5 min 31 s) performed at six b-values (0, 50, 100, 200, 400, and 800 s/mm^2^; gradient system: strength 45 mT/m, slew rate 200 T/m/s)). The high b-value of 800 s/mm^2^ was chosen in line with consensus recommendations for breast DWI (22). ADC maps were generated by the scanner software after DWI acquisition. This step was followed by a 3D non-selective inversion recovery -prepared spoiled gradient echo sequence (repetition time/echo time: 2.8/0.93 ms, flip angle: 8°, field of view: 340×340×180 mm, matrix size: 128×128×36, slice thickness: 5 mm, acceleration factor: 2, inversion recovery - repetition time: 3000 ms, overall acquisition time: 4 min 20 s), performed at four inversion times (100, 600, 1200 and 2800 ms) to estimate T_1_. Both breasts, the aortic arch and part of the descending aorta were included in the field of view (21).

Afterwards, interleaved high temporal resolution (HTR) and high spatial resolution (HSR) DCE-MRI sequences were employed (5). The dynamic series consisted of 93 HTR images interleaved with 8 HSR images acquired as follows (10×HTR, 1×HSR, 43×HTR, [1×HSR, 5×HTR] repeated seven times, and finally 5×HTR). The HTR dynamic images were acquired using a T_1_-weighted 3D spoiled gradient echo sequence (repetition time/echo time: 2.37/0.73 ms, flip angle: 25°, field of view: 340×340 ×180 mm, matrix size: 128×128×36, slice thickness: 5 mm, acceleration factor: 2×2, acquisition time: 2 s). For the HSR images, a fat-suppressed T_1_-weighted 3D spoiled gradient echo sequence (repetition time/echo time: 4.1/1.2 ms, flip angle: 10°, field of view: 340×340 ×180 mm, matrix size: 384×384×128, slice thickness: 1.4 mm, acceleration factor: 3, and acquisition time: 36 s) was employed. The HTR and HSR images were acquired with the same geometry as the inversion recovery sequence. Using an automated power injector (Spectris Solaris EP), gadolinium-based contrast agent (Dotarem, Guerbet Laboratories) was administered intravenously (0.1 mmol/kg) at the start of the eleventh HTR-DCE-MRI image, followed by saline (20 ml at a rate of 3 ml/s). A second inversion recovery T1 estimate (bookend) was performed (23) after all eight HSR (and 88 HTR images) images were obtained. Then, the last five HTR images were acquired.

### 2.3 Image analysis

**Figure 1:**
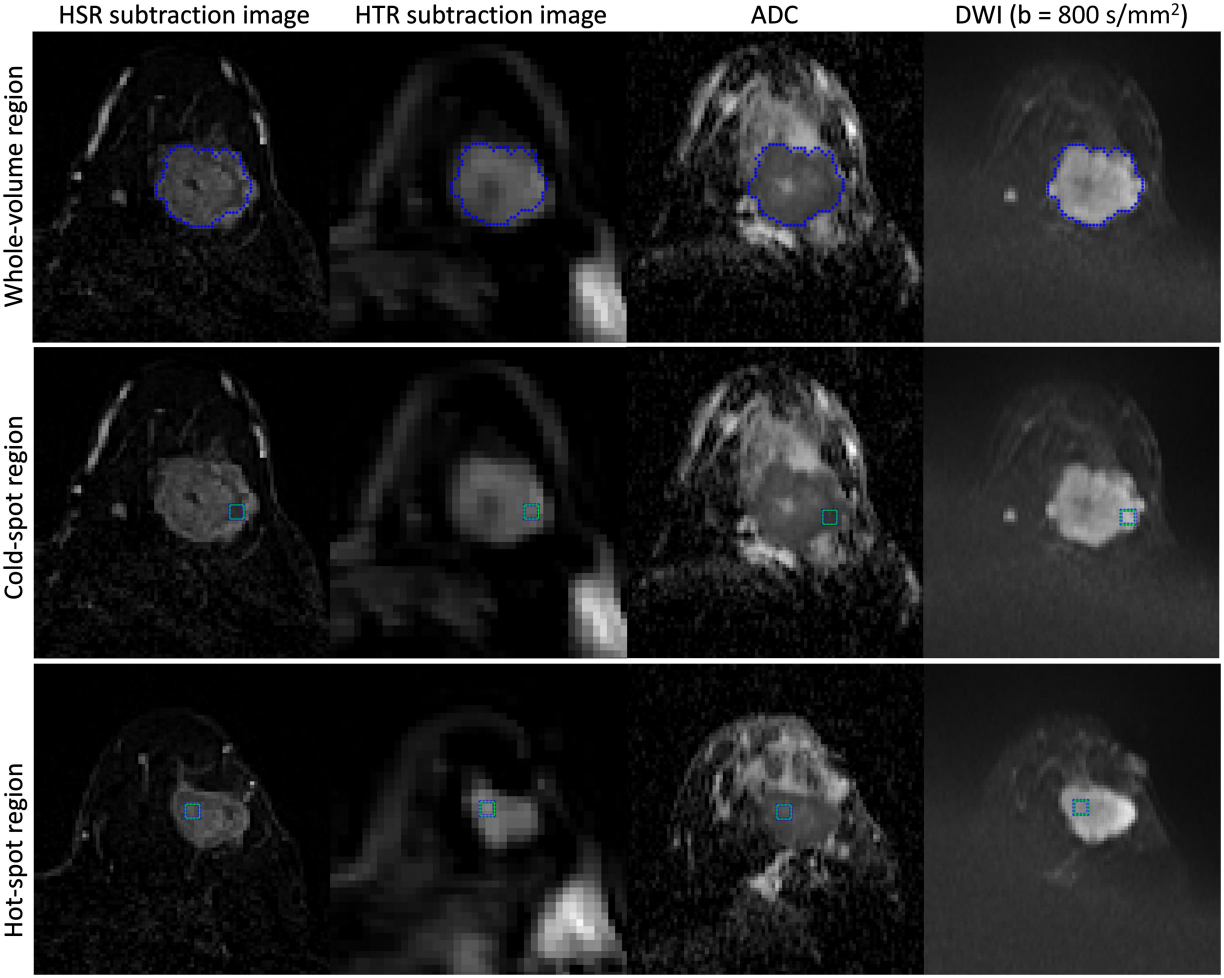
Example of seeding a tumor in a 45-year-old woman with invasive ductal carcinoma in the left breast and generating three regions of interest (whole-tumor, cold-spot, and hot-spot). First, the tumor was seeded on the HSR subtraction images, and the whole-tumor region of interest was generated (top row). Then, two smaller regions of interest (5×5 pixels) were generated within the whole-tumor region (cold-spot (middle row) and hot-spot regions (bottom row)). The whole-tumor region of interest encompasses all the slices in which the tumor appears, while the cold-spot and hot-spot regions originate in only a single slice (not necessarily the same slice). All three regions were propagated to the corresponding DWI, inversion recovery, and HTR images for further analysis.

MRI data were processed using in-house programs developed in MATLAB (MathWorks, USA). The DWI images (including ADC maps) were rigidly aligned to the corresponding HSR, HTR and inversion recovery images to match the slice position with no interpolation of the DWI data, and HTR and HSR subtraction images were generated to improve tumor visibility. The location of the largest tumor for each patient was determined using HSR DCE-MRI images from the baseline MRI, confirmed by a breast radiologist. Then, a whole-tumor region of interest was generated using a 3D region-growing algorithm based on the enhanced tumor’s signal intensity in HSR subtraction images, while avoiding obvious necrotic areas manually. Two smaller single-slice regions of interest (5×5 pixels) within the whole-tumor region were generated to reduce the possibility of tumor heterogeneity compromising subsequent correlation analysis. These small regions comprised the region with the lowest ADC on the ADC map (cold-spot region) (22) and the region with the highest SI on the HTR subtraction images (hot-spot region). All three regions were propagated to the corresponding DWI, inversion recovery and HTR images for further analysis (**Figure 1**). The spatial location of the smaller regions generated for each tumor were allowed to vary at each MRI visit as the tumor responded to NACT.

For DCE-MRI, these three regions were used to estimate T1 relaxation-times from both sets of inversion recovery images. A further region of interest was drawn in the descending aorta to generate signal intensity -time curves and estimate T1 before and after gadolinium-based contrast agent injection for measurement of the arterial input function (21). The signal intensity -time data were converted to gadolinium-based contrast concentration-time using a bookend T1 correction with an iterative scheme (21, 23). A two–compartment exchange model was fitted to the DCE-MRI data, and tumor blood flow, blood volume fraction, capillary permeability–surface area product and interstitial volume fraction were estimated (24). Then, the extracellular volume fraction (the sum of interstitial and blood volume fractions) was calculated. For each region of interest, a tissue uptake model (described by parameters blood flow, blood volume fraction, and capillary permeability–surface area product) and a one–compartment model (described by parameters blood flow and extracellular volume fraction) were also fitted to the DCE-MRI data (24). The final model to use in the correlation analysis was selected based on the corrected Akaike information criterion test (cAIC) to evaluate which model best fits the data (25, 26).

For DWI, the mean signal intensity for each b−value was extracted from the three regions (27). The IVIM parameters were estimated by fitting the bi-exponential model to the mean signal intensity vs b-value data using an over-segmented approach, where tissue diffusion and perfusion fraction were estimated first and then pseudo-diffusion (28). The monoexponential model was also fitted to the mean signal intensity vs b-value, and the ADC value for each region of interest was estimated (28). The two model equations are detailed in the **Supplementary Material** (Appendix A). This step was conducted blinded to the DCE-MRI parameter values. Further, a simulation study was performed to assess the bias and precision of the IVIM parameter estimates with 6 b-values in comparison with 12 b-values (methods and results are provided in Appendix B, **Supplementary Material**).

### 2.4 Statistical analysis

Due to the non-normal data distribution, the DWI and DCE-MRI data were summarized using the median (interquartile range). Friedman’s test with Bonferroni correction (Bonferroni *post hoc* test) was performed for each parameter from the baseline MRI to determine whether parameter differences existed between the three regions of interest (whole-tumor, cold-spot, and hot-spot). To determine the between-subject correlation between IVIM and DCE-MRI parameters for each region, the mean value of each parameter for each patient was calculated by dividing the sum of parameter values from all MRI visits by the number of times the parameter was estimated; then, the parameter value for each visit where the parameter was estimated was replaced by its subject mean. The weighted correlation coefficient, r, was calculated between the mean DWI and DCE-MRI parameters for each region of interest using the Spearman’s rank correlation test (29) (r<0.2, very weak; 0.2≤r<0.4, weak; 0.4≤r<0.7, moderate; 0.7≤r<0.9, strong; r≥0.9, very strong correlation) (30). This statistical method was followed to exploit the properties of data with multiple measures while addressing the issue of non-independence among observations and the impact of NACT (29). Statistical analyses were performed using SPSS software for Windows (v.25.0, Chicago, IL). All tests were two-sided, and a p-value of less than 0.05 was considered statistically significant.

To determine the correlation between changes in the IVIM and DCE-MRI parameters induced by treatment, the repeated measures correlation test (rmcorr) was utilized via the rmcorr-shiny app (31, 32). The rmcorr-shiny app computes a repeated measures correlation coefficient (r_rm_) that considers the dependence between repeated measurements. This analysis involves determining the correlation between two parameters while accounting for between-subject variation. The rmcorr-shiny app fits separate parallel lines to each patient’s data utilizing a shared slope but permitting the intercept to differ per patient. The orientation of these parallel lines represents the correlation’s sign (positive or negative), while the slope denotes the correlation’s magnitude.

The results of repeated measures correlation for each region were summarized in tables as: r_rm,_ degrees of freedom, 95% confidence interval, and a p-value. The 95% confidence interval for each r_rm_ were determined using bootstrapping with 1000 resamples. The degrees of freedom (df) were computed based on the formula df = N(k-1) – 1, where N is the total number of patients and k is the (average) number of repeated measures per patient (31). The rmcorr test was initially conducted to identify statistically significant results (P-value < 0.05), then bootstrapped 95% confidence intervals were calculated. A correlation result was considered meaningful and significant only if the magnitude of the correlation coefficient was ≥ 0.4, the P-value was less than 0.05, and the bootstrapped 95% confidence intervals excluded zero. Since this is a preliminary exploration study focusing on hypothesis generation, P-values for the correlation tests were reported as raw values and were not corrected for multiple comparisons. An upper estimate of the repeatability of the DWI and DCE-MRI parameters was calculated from a subset of baseline and cycle 1 studies (details included in Appendix C, **Supplementary Material**).

## 3 Results

In this study, 40 female patients were eligible and enrolled between August 2015 and April 2018 (median age 44.5 (39, 53) years). MRI data were obtained for all patients at baseline and 37 patients after one and three cycles of NACT (three withdrew following baseline MRI). However, the MRI data acquired after three NACT cycles from two patients were excluded from the analysis because no tumor was apparent on their MRI scans. This exclusion resulted in 112 MRI studies with DWI and DCE-MRI acquisitions. **Table 1** presents the clinical characteristics of the patients.

**Table 1:** Clinical characteristics of all the enrolled patients.

Characteristic	Number or Median (Interquartile range)
Number of patients	40
Age (years)	44.5 (38.8, 53.0)
Tumor volume (cm^3^)	
At baseline (N= 40)	5.45 (2.16, 16.27)
After one cycle of NACT (N= 37)	4.1 (1.57, 8.83)
After three cycles of NACT (N= 35)	2.15 (0.53, 5.8)
Tumor grade	
II	15
III	25
Tumor type	
Invasive ductal carcinoma	38
Inflammatory breast cancer	1
Mucinous carcinoma	1
Estrogen receptor status	
Positive (+)	28
Negative (-)	12
Progesterone receptor status	
Positive (+)	18
Negative (-)	20
Not evaluable	2
Human epidermal growth factor 2 status	
Positive (+)	15
Negative (-)	25

**Figure 2:**
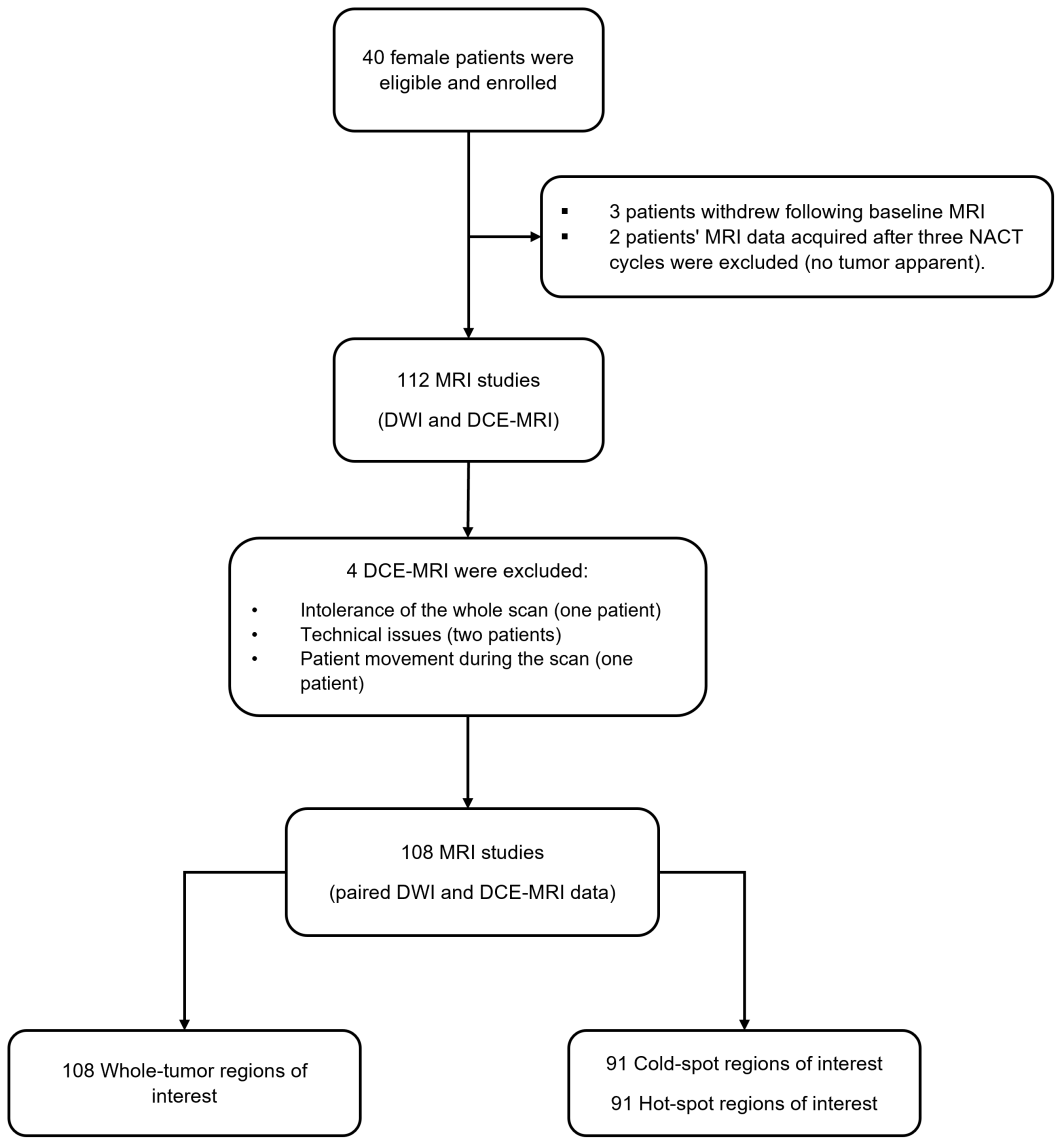
The flow chart illustrates the number of recruited patients, excluded DCE-MRI data, and the final number of MRI studies with paired DWI and DCE-MRI data acquisitions.

Four DCE-MRI scans—two at baseline and two after three NACT cycles—were excluded because one patient could not tolerate the whole imaging protocol, two had technical issues (the back coil was switched on and off sporadically), and one moved during the DCE-MRI acquisition, leaving 108 studies with paired DWI and DCE-MRI data acquisitions (**Figure 2**). Based on the cAIC results, 75 DCE-MRI data sets were analyzed using the two–compartment exchange model, 20 using the tissue uptake model, and 13 using the one–compartment model.

Smaller regions of interest (cold-spot and hot-spot regions) were generated from 91 out of 108 studies: 34 at the baseline, 33 after one NACT cycle, and 24 after three NACT cycles (some tumors shrank below the 5x5 pixels threshold during NACT). DCE data sets were fitted using the two-compartment exchange/tissue-uptake/one-compartment models for 68/13/10 cold-spot regions and for 72/9/10 hot-spot regions.

For DWI data analysis, there were a number of cases where estimates of the IVIM parameters pseudo-diffusion and perfusion fraction reached one of their limiting values, and these parameters were excluded from the statistical analyses (2 cases from the whole-tumor region, 8 from the cold-spot region, and 4 from the hot-spot region).

### 3.1 Estimated DWI and DCE-MRI parameters from the three regions at baseline

There were significant differences between the parameter values estimated in whole-tumor, cold-spot, and hot-spot regions for all DWI and DCE-MRI parameters, with the exception of pseudo-diffusion and extracellular volume fraction (P=0.88 and 0.2, respectively). Detailed results, including pairwise comparisons (Bonferroni-corrected), are presented in the **Supplementary Material** (**Supplementary Table A1**).

### 3.2 Correlation between averaged DWI and DCE-MRI parameters from three MRI visits (between-subject correlation)

**Figure 3:**
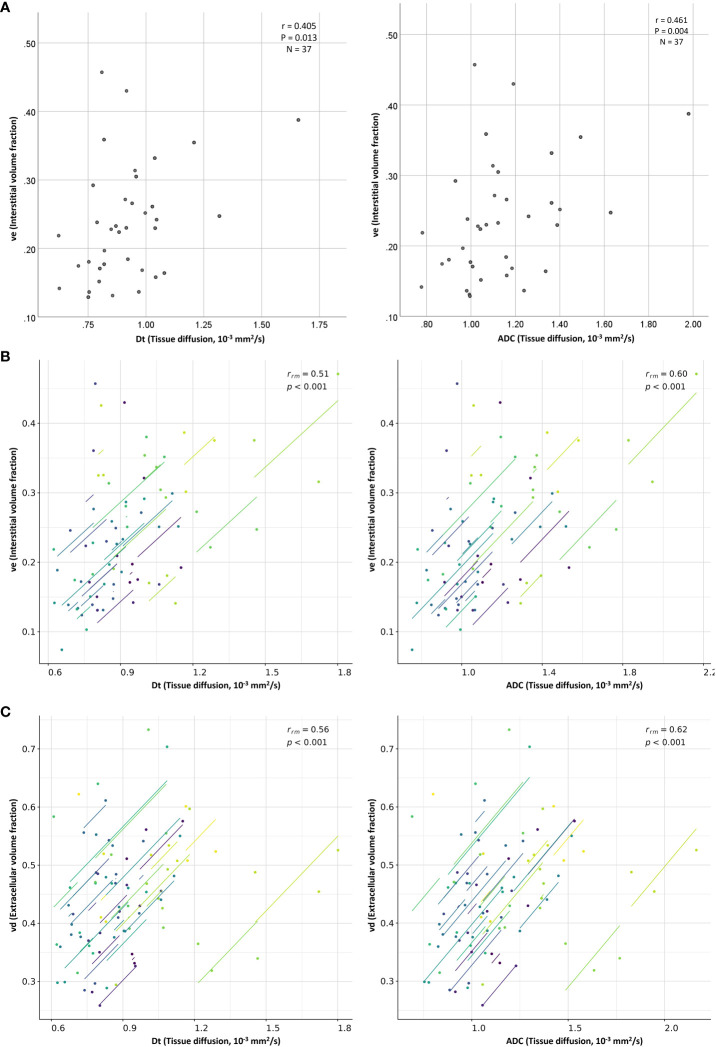
Scatter plots show moderate positive **(A)** between-subject and **(B)** within-subject repeated measures correlations between the diffusion coefficients (ADC and D_t_) and the interstitial volume fraction (v_e_), as well as moderate positive **(C)** within-subject repeated measures correlation between the diffusion coefficients (ADC and D_t_) and the extracellular volume fraction (v_d_). Each line in the scatter plots (**B**, **C**; repeated measures correlations) shows the fit for a single patient.

No significant correlations were discovered between the IVIM and DCE-MRI perfusion-related parameters (perfusion fraction with blood volume fraction, and microvascular blood flow with blood flow) in the three tumor regions (P=0.146–0.379, **Table 2**, **Supplementary Table A2**, **Supplementary Table A3**). However, for whole-tumor regions, ADC exhibited a significant moderate positive correlation with tumor T_1_ and interstitial volume fraction (r = 0.603 and 0.461, respectively). Similarly, D_t_ demonstrated a significant moderate positive correlation with tumor T_1_ and interstitial volume fraction (r = 0.631 and 0.405, respectively). (**Figure 3**, **Table 2**).

**Table 2:** Correlation between averaged DWI and DCE-MRI parameters from three MRI visits (Whole-tumor region).

Parameter		Tumor T_1_	F_b_	PS	v_e_	v_b_	v_d_
ADC	**r**	0.603^**^	0.026	0.305	0.461^*^	-0.173	0.302
	**P-value**	**<0.001**	0.873	0.056	**0.004**	0.286	0.058
	**N**	**40**	40	40	**37**	40	40
D_t_	**r**	0.631^**^	0.014	0.266	0.405^*^	-0.135	0.302
	**P-value**	**<0.001**	0.932	0.097	**0.013**	0.406	0.058
	**N**	**40**	40	40	**37**	40	40
D_p_	**r**	-0.251	0.172	-0.051	-0.360	-0.006	-0.213
	**P-value**	0.118	0.289	0.755	0.029	0.971	0.187
	**N**	40	40	40	37	40	40
f	**r**	0.187	0.121	0.186	0.093	-0.144	0.079
	**P-value**	0.248	0.457	0.251	0.584	0.375	0.628
	**N**	40	40	40	37	40	40
f×D_p_	**r**	-0.020	0.143	0.071	-0.126	-0.041	0.001
	**P-value**	0.903	0.379	0.663	0.457	0.802	0.995
	**N**	40	40	40	37	40	40

r, correlation coefficient; N, sample siz;. ADC, apparent diffusion coefficient; D_t_, tissue diffusion; D_p_, pseudo-diffusion coefficient; f, perfused fraction; f×D_p_, microvascular blood flow; F_b_, blood flow; PS, capillary permeability–surface area product; v_e_, interstitial volume fraction; v_b_, blood volume fraction; v_d_, extracellular volume fraction.

Values in bold indicate significant correlation results: * r ≥ 0.4 and P<0.05.

** r ≥ 0.4 and P<0.001.

In the cold-spot regions, significant moderate positive correlations were found between tumor T_1_ and both measures of tissue diffusion ADC and D_t_ (r= 0.632 and 0.588, respectively). pseudo-diffusion demonstrated a significant moderate negative correlation with blood flow (r = -0.400, **Supplementary Table A2**). In hot-spot regions, ADC and D_t_ displayed significant moderate positive correlations with tumor T_1_ (r=0.520 and 0.460, respectively, **Supplementary Table A3**).

### 3.3 Repeated measures correlations between DWI and DCE-MRI parameters (within-subject correlation)

**Table 3** lists the repeated measures correlation results computed between the DWI and DCE-MRI parameters estimated from the whole-tumor regions of interest. No statistically significant correlations were discovered between the IVIM and DCE-MRI perfusion-related parameters of the study’s primary interest (perfusion fraction versus blood volume fraction and microvascular blood flow versus blood flow; P=0.815 and 0.229, respectively). However, ADC and D_t_ displayed significant moderate positive correlations with interstitial volume fraction (r_rm_=0.597 and 0.514, respectively) and extracellular volume fraction (r_rm_=0.619 and 0.564, respectively) (**Figure 3**).

**Table 3:** Repeated measures correlations between DWI and DCE-MRI parameters estimated from Whole-tumor region.

Parameter		Tumor T_1_	F_b_	PS	v_e_	v_b_	v_d_
ADC	**r_rm_**	0.035	-0.361	-0.138	0.597^**^	0.226	0.619^**^
	**df**	67	67	54	**37**	54	**47**
	**P-value**	0.775	0.002	0.309	**<0.001**	0.094	**<0.001**
	**95% CI**	-0.18, 0.253	-0.605, 0.01	-0.452, 0.253	**0.203, 0.785**	-0.012, 0.432	**0.383, 0.82**
D_t_	**r_rm_**	0.043	-0.32	-0.045	0.514^**^	0.165	0.564^**^
	**df**	67	67	54	**37**	54	**47**
	**P-value**	0.724	0.007	0.741	**<0.001**	0.224	**<0.001**
	**95% CI**	-0.217, 0.279	-0.544, 0.036	-0.339, 0.312	**0.103, 0.716**	-0.052, 0.373	**0.305, 0.785**
D_p_	**r_rm_**	0.125	0.336	0.157	-0.127	-0.208	0.078
	**df**	65	65	53	37	53	46
	**P-value**	0.313	0.005	0.253	0.442	0.127	0.597
	**95% CI**	-0.08, 0.268	0.092, 0.502	-0.08, 0.397	-0.311, 0.074	-0.402, -0.02	-0.221, 0.304
f	**r_rm_**	0.04	-0.182	-0.237	0.354	0.165	0.297
	**df**	65	65	53	37	53	46
	**P-value**	0.748	0.139	0.081	0.027	0.229	0.04
	**95% CI**	-0.201, 0.229	-0.423, 0.137	-0.477, 0.034	0.017, 0.583	-0.19, 0.418	0.068, 0.509
f×D_p_	**r_rm_**	0.055	0.029	-0.059	0.215	-0.035	0.252
	**df**	65	65	53	37	53	46
	**P-value**	0.661	0.815	0.668	0.188	0.799	0.084
	**95% CI**	-0.178, 0.228	-0.169, 0.265	-0.222, 0.123	-0.066, 0.444	-0.261, 0.187	-0.023, 0.445

r_rm_, repeated measures correlation coefficient; df, degrees of freedom; CI, confidence interval; ADC, apparent diffusion coefficient; D_t_, tissue diffusion; D_p_, pseudo-diffusion coefficient; f, perfused fraction; f×D_p_, microvascular blood flow; F_b_, blood flow; PS, capillary permeability–surface area product; v_e_, interstitial volume fraction; v_b_, blood volume fraction; v_d_, extracellular volume fraction.

Values in bold indicate significant correlation results: * r_rm_ ≥ 0.4, P<0.05, and bootstrapped 95% CIs excluded zero.

** r_rm_ ≥ 0.4, P<0.001 and bootstrapped 95% CIs excluded zero.

**Figure 4:**
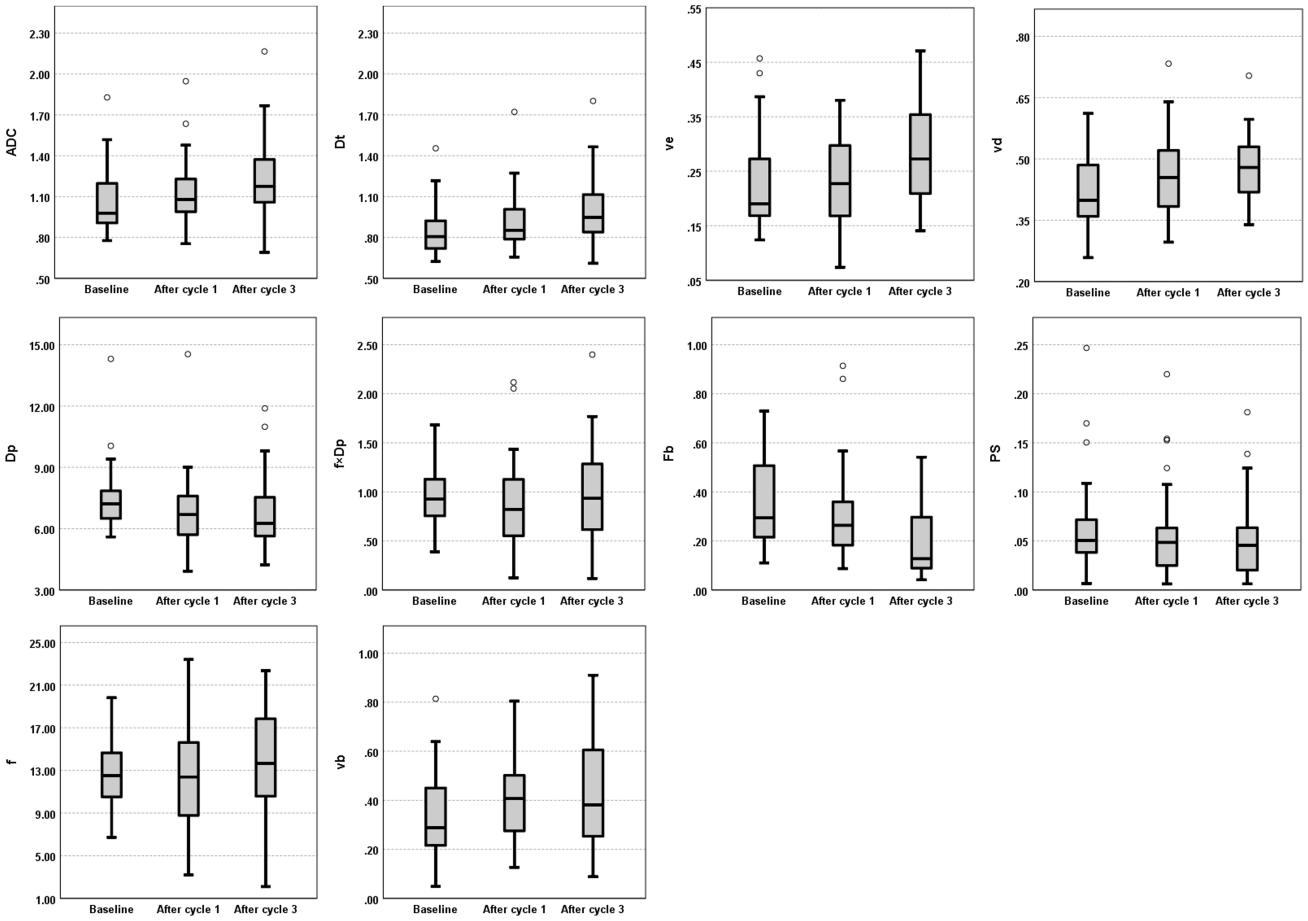
Evolution of DWI (ADC, D_t_, D_p_, f, and f×D_p_) and DCE-MRI (F_b_, v_b_, PS, v_e_, and v_d_) parameters across the three MRI visits (Baseline, and after one and three cycles of NACT). Box plots illustrate the median and interquartile range values of all patients for whole-tumor region at each MRI visit. ADC, apparent diffusion coefficient; D_t_, tissue diffusion; v_e_, interstitial volume fraction; v_d_, extracellular volume fraction; D_p_, pseudo-diffusion coefficient; f×D_p_, microvascular blood flow; F_b_, blood flow; PS, capillary permeability–surface area product; f, perfused fraction; v_b_, blood volume fraction.

The median DWI and DCE-MRI parameter values estimated at the three MRI visits from the cold-spot and hot-spot regions exhibited patterns similar to those of the whole-tumor regions but with much more variability (**Figure 4**, **Supplementary Figure A1**); repeated measures correlation results in the cold-spot and hot-spot regions are presented in **Supplementary Data** only (**Supplementary Table A4**, **Supplementary Table A5**).

## 4 Discussion

Despite the examination of 108 paired DWI and DCE-MRI datasets, no statistically significant between-subject or within-subject repeated measures correlations were found between the IVIM and DCE-MRI perfusion parameters of the study’s primary interest (perfusion fraction versus blood volume fraction and microvascular blood flow versus blood flow). These findings align with previous breast cancer studies, which also found no correlation between any IVIM perfusion parameters and DCE-MRI parameter related to perfusion, K^trans^ (transfer constant) (18, 19). K^trans^ may not solely reflect tumor blood flow but also vessel permeability (33). The present study went further by estimating tumor blood flow and blood volume fraction from DCE-MRI during NACT, but still found no correlations. One possible explanation for the lack of correlation might be significant tissue heterogeneity in tumors; the parameters were estimated from the whole-tumor regions. Where possible, two smaller regions of interest (5×5 pixels) in each whole-tumor region were generated to reduce the likelihood of heterogeneity. It was assumed that these smaller regions would be more homogenous. However, no clear correlations were found in these smaller regions, and the data were observed to be more variable than the whole-tumor region, as reflected by the number of outliers and a wider range in the box plot scale (**Figure 4**, **Supplementary Figure A1**). An alternative method for future studies that might aid in selecting homogeneous tumor regions could be histogram analysis of pixel-wise IVIM and DCE-MRI parameter maps; however, the possibility of finding a homogeneous tumor region in the IVIM and DCE perfusion-related parameter maps to examine the correlation would require further investigation and validation.

Imprecision in the estimates of microvascular blood flow and blood volume fraction, in particular, is a potential issue that may have masked correlations between the IVIM and DCE-MRI perfusion parameters. A previous report recognized that the precision with which pseudo-diffusion is estimated is poor (34), and the estimate of blood volume fraction in another study was reported to be very imprecise (35), which was reflected in our calculated upper estimate of its repeatability (Appendix C, **Supplementary Material**). The estimation of blood volume fraction, against which the perfusion fraction derived from IVIM is compared, becomes difficult when tumor capillaries are excessively leaky (24). In this study, out of 108 DCE-MRI datasets, a one-compartment model was preferred in 13 cases, and an estimate of blood volume fraction and capillary permeability–surface area product was not possible in those 13.

It is also possible that IVIM and DCE-MRI reflect different underlying physiology. IVIM does not estimate perfusion in a classical way but estimates flow in the direction of the diffusion encoding gradient, whereas DCE-MRI measures the delivery of blood and subsequent distribution of contrast agent in the tissue, on a different time scale (36). Furthermore, it has been suggested that a single pseudo-diffusion coefficient is insufficient to describe the complex diffusion properties of the vascular signal (37). The inconsistent patterns of response to treatment seen in the median values of perfusion fraction versus blood volume fraction and microvascular blood flow versus blood flow may support this suggestion (**Figure 4**).

In contrast, this study found moderate positive between-subject and within-subject repeated measures correlations between the diffusion parameters (ADC and D_t_) and interstitial volume fraction, as well as a moderate positive within-subject repeated measures correlation between the diffusion parameters and extracellular volume fraction. These positive results are important, as this is the first time they have been observed in breast cancer (8), and support the current understanding of these imaging parameters. A positive between-subject correlation between ADC and interstitial volume fraction was previously determined in head and neck cancers (9) suggesting that these parameters are related to tissue microstructure. The ADC and D_t_ values reflect the diffusion of water molecules in tissue, which is affected by cellular density, membrane permeability and extracellular volume (7), and v_d_ is a direct measure of the extracellular volume fraction (24) while v_e_ is a parameter that reflects the volume fraction of the interstitial space within the tissue, which can be influenced by such factors as cellular density and extracellular matrix deposition. A prior study revealed that tumor cellularity is inversely proportional to v_e_, v_d_, and ADC values (38). Therefore, the observed between-subject correlation of the diffusion coefficients and interstitial space may suggest that breast tumors with a high cellular density tend to have a small interstitium and increased diffusion restriction, whereas tumors with a low cellular density tend to have a large interstitium and less diffusion restriction. The observed positive within-subject repeated measures correlations could result from the fact that ADC/D_t_, v_e_, and v_d_ exhibited similar patterns of change in response to treatment, wherein the values were increasing during the three MRI time-points (**Figure 4**).

Furthermore, a moderate positive between-subject correlation between the diffusion coefficients and tumor T_1_ was observed in this study. Tumor T_1_ measures tissue relaxation time, which can be affected by tissue water and fat content, macromolecule concentration and hydration state (39). Thus, this positive correlation may be because breast tumors with high cellular density and a small extracellular space have a decreased free-water content, resulting in low diffusion coefficient values and short tumor T_1_ (12, 39).

The present study has some limitations. First, this study was performed on a limited sample size using a 1.5 T MRI scanner, which may limit the statistical power of the results. However, this is the first study that assesses both between-subject and within-subject repeated correlations between the perfusion parameters estimated by IVIM and DCE-MRI in a cohort of breast cancer patients undergoing NACT with a primary focus on hypothesis generation rather than testing; therefore, the results can be used to direct future studies. Second, the DWI data were acquired with only 6 b-values, four of which were low (≤ 200 s/mm^2^). In a clinical protocol, it is not practical to acquire DWI data with a large number of b-values. Nevertheless, the simulation study showed that using 6 b-values will not result in appreciably worse outcomes for most parameters, though the precision of microvascular blood flow was lower than with 12 b-values (details provided in Appendix B, **Supplementary Material**). Further, a previous study showed that a small number of b-values is not the main source of errors in IVIM parameter estimates. Intra-patient variability is significant; they found that the precision in the estimates of the IVIM parameters with only 4 b-values was better than the test-retest repeatability of those same parameters estimated with 16 b-values (34). Third, a pixel-wise comparison of IVIM and DCE-MRI parameter maps was not performed in this study, although it might be valuable. Instead, the images were analyzed by following the recommended approaches of the International Breast Diffusion-Weighted Imaging Working Group (22), which included volumetric sampling and focused regions of interest (i.e., smaller single-slice regions on the darkest part of the ADC map). No correlations were observed between perfusion fraction versus blood volume fraction and microvascular blood flow versus blood flow in these smaller regions, but they showed more variability in the estimates instead (**Supplementary Figure A1**), suggesting that a pixel-wise analysis might yield similar outcomes. Fourth, rigid registration was employed for aligning the DCE and DWI images and this approach may not have been sufficient to correct DWI distortions. As such, the accuracy of spatial co-registration could have been affected, potentially influencing the findings reported, particularly in the smaller regions. Therefore, future work incorporating pixel-wise analysis following rigorous DWI and DCE-MRI image registration is needed to further investigate these relationships. Finally, the repeatability of the DWI and DCE-MRI parameters was not formally investigated. It was challenging to justify performing a repeated baseline DCE-MRI scan that required an additional injection of gadolinium contrast because the patients were due to undergo multiple NACT cycles and MRI scans. Instead, an upper estimate of the repeatability of the DWI and DCE-MRI parameters was calculated from a selection of baseline and cycle 1 studies (Appendix C, **Supplementary Material**).

In conclusion, this preliminary study investigated both between-subject and within-subject repeated measures correlations between DWI and DCE-MRI parameters in a cohort of patients with breast cancer imaged before and after one and three cycles of NACT. No statistically significant correlations were observed between the perfusion parameters estimated by IVIM (perfusion fraction and microvascular blood flow) and those estimated by DCE-MRI (blood flow and blood volume fraction). The two techniques may reflect different underlying physiology, and/or estimates of the IVIM and DCE-MRI parameters in the current study are largely imprecise. Therefore, care should be taken when interpreting the IVIM perfusion parameters as surrogates for those measured using DCE-MRI until their underlying pathophysiologic interpretation and relationship to the DCE-MRI perfusion parameters are elucidated by further research. However, the moderate positive within-subject repeated measures correlations found between the diffusion parameters and DCE-MRI measures of the tissue’s interstitial and extracellular volume fractions confirms the expectation that as these volumes increase, water diffusion increases.

## Data availability statement

The raw data supporting the conclusions of this article will be made available by the authors, without undue reservation.

## Ethics statement

The studies involving humans were approved by Yorkshire & The Humber - Bradford Leeds Research Ethics Committee (Ethics approval: 15/YH/0246). The studies were conducted in accordance with the local legislation and institutional requirements. The participants provided their written informed consent to participate in this study.

## Author contributions

ZA: Writing – review & editing, Writing – original draft, Visualization, Validation, Software, Methodology, Investigation, Formal Analysis, Data curation. SB: Writing – review & editing, Software, Methodology. DW: Writing – review & editing, Supervision, Funding acquisition. NS: Writing – review & editing, Funding acquisition. TD: Writing – review & editing, Supervision. DB: Writing – review & editing, Validation, Supervision, Software, Resources, Project administration, Methodology, Funding acquisition, Data curation, Conceptualization.
