# Supplementary material for: The relationship between parameters measured using intravoxel incoherent motion and dynamic contrast-enhanced MRI in patients with breast cancer undergoing neoadjuvant chemotherapy: a longitudinal cohort study
Source: Front Oncol. 2024 May 22;14:1356173. doi: 10.3389/fonc.2024.1356173 (PMC11163445; doi:10.3389/fonc.2024.1356173)
Supplement: Supplementary file 1 [file DataSheet_1.pdf]

SUPPLEMENTARY MATERIAL

Appendix A: Supplementary Methods, Tables and Figures for the clinical study

1 Supplementary Methods

The monoexponential and IVIM models parameter estimation

The parameter values of the monoexponential and IVIM models were obtained by fitting to the mean signal intensity (SI) against b-value data using a nonlinear least-squares approach with the following equations:

1. Monoexponential model:

The monoexponential model was fitted to the mean SI vs b-value data using the nonlinear least-squares approach with the following equation:

$$S(b) = S(0).exp(-b\,.ADC) \qquad \text{Equation (1)}$$

S(b) is the mean SI obtained with a b-value of b. Following the ACRIN 6698 multicentre trial (1), S(0) and the apparent diffusion coefficient (ADC) values were simultaneously calculated from a monoexponential fit using all b-value data.

2. Bi-exponential IVIM model:

The bi-exponential model was fitted to the mean SI vs b-value data using the over-segmented approach with the following equation:

$$S(b) = S(0).\ [(1-f)\,exp(-b\,.\,Dt) + f\ exp(-b\,.\,Dp)]$$

f is the perfused fraction representing the pseudo-diffusion fraction associated with microcirculation. D_t reflects the tissue diffusion coefficient, and D_p reflects the pseudo-diffusion coefficient. The three parameters were consecutively calculated. The D_t value was estimated first by fitting to b-value data (400 and 800 s/mm^2) using a monoexponential fit as in Equation (A.1), with the following bound constraints ($0 \le D_t \le 5 \times 10^{-3}$ mm^2/s). This estimate assumes that the influence of D_p on the diffusion-weighted signal can be neglected when the b-value ≥400 s/mm^2. Second, the monoexponential fit was extrapolated back to b=0 to estimate f as f = $(S(0)_{\text{"measured"}} - \text{intercept})/S(0)_{\text{"measured"}}$, where “intercept” is the fitted S(0) found when estimating D_t. Finally, by fixing D_t and f values, D_p was obtained using a nonlinear least-squares fitting approach with the following constraints ($Dt < Dp \le 100 \times 10^{-3}$ mm^2/s) (2). If the estimate of f < 0, f is forced to be 0, and D_p is not estimated, and the fitting result will be considered a monoexponential.

Appendix B: Simulation study description and results

This study was performed in Matlab using simulated signal data generated according to the IVIM model using 14 different parameter sets of S(0), f, D_t, D_p, $f \times D_p$ and that represent the full range of IVIM parameters seen in the previous study on the same subjects (3) (Table B1). For each IVIM parameter value set, the signals were sampled at 12 b-values (b= 0, 10, 20, 30, 50, 70, 100, 150, 200, 400, 800, 1000 s/mm^2). Then, Rician noise was added to the sampled signals (i.e., 12 b-values) to achieve a noise level similar to that seen in the subjects’ data.

The noisy signals were fitted, and the fitting process was performed using the 6 b-values used in the present study (0, 50, 100, 200, 400, 800 s/mm^2). Each simulation trial was repeated 1000 times.

Table B1. True value sets of IVIM parameters for simulations study

**No.**	**S(0)**	**D_t (×10^{-3} mm^2/s)**	**D_p (×10^{-3} mm^2/s)**	**f (no units)**	**f×D_p (×10^{-3}mm^2/s)**
1	200	1.08	6.10	0.213	1.30
2	200	1.21	5.57	0.208	1.15
3	200	1.20	6.80	0.197	1.34
4	200	0.98	6.47	0.184	1.19
5	200	0.90	5.95	0.174	1.03
6	200	1.01	5.45	0.164	0.89
7	200	0.95	6.47	0.154	1.00
8	200	0.90	6.46	0.145	0.94
9	200	0.81	5.97	0.132	0.78
10	200	0.91	5.59	0.126	0.70
11	200	0.79	6.49	0.114	0.74
12	200	0.70	6.53	0.107	0.69
13	200	0.82	8.08	0.093	0.75
14	200	0.68	7.23	0.081	0.59

S(0): the signal intensity with b-value of zero. D_t: tissue diffusion. D_p: pseudo-diffusion coefficient. f: perfused fraction. f×D_p: microvascular blood flow.

Then, the relative bias in the estimation of the parameters D_t, f, and f×D_p was calculated in comparison to their true values (4) (Table E6), as follows:

- Relative bias (RB) = $\frac{(\textit{Fitted parameter value} - \textit{True parameter value})}{\textit{True parameter value}}$

The standard deviation (SD) of the 1000 fits for each parameter was calculated. Finally, relative bias and standard deviation calculated from the 14 simulated data sets were summarized as mean (95% confidence interval) according to the number of b-values. This work was performed with the aim of evaluating the effect of the number of b-values on the bias and precision of IVIM parameters (D_t, f, and f×D_p) which are of primary interest in assessing correlations with DCE-MRI parameters (F_b, v_b, v_e, and v_d).

The results showed that acquiring DWI data with 6 b-values will not lead to appreciably biased and imprecise results for the IVIM parameter estimates (D_t and f) compared to acquiring the same data with 12 b-values, though the precision of f×D_p was clearly lower when using 6 b-values (SD in Table B2).

Tabel B2. Comparisons of the mean relative bias and standard deviation of IVIM parameters among 6 and 12 b-values in the simulation study.

Measures	Parameter	6 b-values Mean (95% CIs)	12 b-values Mean (95% CIs)
RB	D_t	0.050 (0.040, 0.060)	0.040 (0.031, 0.048)
	f	-0.228 (-0.263, -0.194)	-0.201 (-0.232, -0.169)
	$f \times D_p$	0.009 (-0.008, 0.026)	0.010 (-0.0004, 0.019)
SD	D_t ($\times 10^{-3}$ mm^2/s)	0.057 (0.052, 0.061)	0.039 (0.035, 0.043)
	f (no units)	0.028 (0.026, 0.029)	0.022 (0.021, 0.024)
	$f \times D_p$ ($\times 10^{-3}$ mm^2/s)	0.194 (0.177, 0.212)	0.131 (0.125, 0.137)

RB: relative bias. SD: standard deviation. CI: confidence interval. D_t: tissue diffusion. f: perfused fraction. D_t: tissue diffusion. f: perfused fraction. $f \times D_p$: microvascular blood flow.

Appendix C: Repeatability analysis and results

An upper estimate of the repeatability of the DWI and DCE-MRI parameters derived from whole-volume ROIs was obtained by calculating the within-subject coefficient of variation (wCV) (5) in the data from baseline and after-one cycle of NACT in a subset of patients who were considered pathological non-responders (pNR) (3) and who showed the smallest changes in tumor volume at cycle one. RECIST guidelines indicate that tumor volume would have to shrink by -66% for a tumor to be considered a partial responder or increase by +73% to be considered progressive disease (6).

Ten pNR were included in the repeatability analysis. The ten tumors showed changes in volume between -21.60% (shrinkage) and +18.90% (increase), with an average volume change of -3.64%.

The wCV for ADC was 4.9%; for the IVIM parameters, it was 5.5% for D_t, 10.9% for D_p, 10.6% for f, and 17.8% for fxD_p; for the DCE-MRI parameters, it was 2.1% for tumor T_1, 13.9% for F_b, 54.8% for PS, 20.6% for v_e, 41.9% for v_b, and 10.2% for v_d. It should be noted that the wCV for v_e and v_d was calculated from 7 pNR patients' data as it was not possible to estimate these parameters in one of the two chosen visits for 3 patients.

2 Supplementary Tables

Table A1. Comparison of DCE-MR and DWI parameter values at baseline for the whole-tumor, cold-spot, and hot-spot regions

**Parameter**	**N**	Whole-volume ROI (a)	Cold-spot ROI (b)	Hot-spot ROI (c)	*P*	Post hoc**
ADC (10^{-3} mm^2/s)	34	**0.98(0.88, 1.20)**	**0.73(0.65, 0.88)**	**0.93(0.86, 1.10)**	**<0.001**	**b<a, b<c**
D_t (10^{-3} mm^2/s)	34	**0.80(0.72, 0.92)**	**0.64(0.59, 0.74)**	**0.81(0.72, 0.93)**	**<0.001**	**b<a, b<c**
D_p (10^{-3} mm^2/s)	25	7.13(6.47, 7.33)	6.94(5.53, 10.18)	7.29(5.69, 8.67)	0.88	-
f (no units)	25	**0.12 (0.11, 0.14)**	**0.09 (0.06, 0.12)**	**0.10(0.08, 0.12)**	**<0.001**	**b<a, c<a**
$f\times D_p$ (10^{-3} mm^2/s)	25	**0.90(0.68, 1.01)**	**0.62(0.43, 0.98)**	**0.71(0.49, 1.00)**	**0.006**	**b<a, c<a**
Tumour T_1 (ms)	34	**1264 (1230, 1322)**	**1252 (1201, 1298)**	**1303 (1256, 1349)**	**<0.001**	**b<c**
F_b (ml/min/ml tissue)	34	**0.29(0.22, 0.55)**	**0.36(0.28, 0.74)**	**0.38(0.27, 0.62)**	**<0.001**	**a<b, a<c**
PS (ml/min/ml tissue)	30	**0.05(0.04, 0.08)**	**0.06(0.04, 0.13)**	**0.09(0.05, 0.17)**	**<0.001**	**a<c, b<c**
v_b (no units)	30	**0.29(0.22, 0.47)**	**0.33(0.23, 0.49)**	**0.3(0.22, 0.44)**	**0.048**	**a<b**
v_e (no units)	24	**0.19(0.16, 0.25)**	**0.17(0.12, 0.22)**	**0.19(0.13, 0.23)**	**0.03**	**b<a**
v_d (no units)	27	0.38(0.35, 0.45)	0.39(0.32, 0.44)	0.36(0.29, 0.46)	0.2	-

Data represented by medians (interquartile ranges). *P* value for a difference between ROIs was found using Friedman's non-parametric test. (The N number differs for the DCE-MRI parameters due to the models used for analyzing the DCE-MRI data, Number of patients= 24-34) Pairwise comparisons** (Bonferroni-corrected) significance at the 0.05 level. ADC: apparent diffusion coefficient. D_t: tissue diffusion. D_p: pseudo-diffusion coefficient. f: perfused fraction. $f\times D_p$: microvascular blood flow. F_b: blood flow. PS: capillary permeability–surface area product. v_b: blood volume fraction. v_e: interstitial volume fraction. v_d: extracellular volume fraction.

Table A2. Correlation between averaged DWI and DCE-MRI parameters from three MRI visits (Cold-spot region)

**Parameter**		Tumour T_1	F_b	PS	v_e	v_b	v_d
ADC	**R**	**0.632****	-0.030	-0.028	0.219	-0.18	0.156
	**P-value**	**<0.001**	0.862	0.871	0.206	0.293	0.371
	**N**	**36**	36	36	35	36	35
D_t	**R**	**0.588****	0.020	0.100	0.283	-0.253	0.115
	**P-value**	**<0.001**	0.908	0.562	0.099	0.137	0.511
	**N**	**36**	36	36	35	36	35
D_p	**R**	-0.139	**-0.400***	-0.126	-0.074	-0.062	-0.149
	**P-value**	0.426	**0.017**	0.471	0.682	0.723	0.4
	**N**	35	**35**	35	33	35	34
f	**R**	0.231	-0.048	0.055	-0.045	-0.202	-0.031
	**P-value**	0.182	0.784	0.754	0.804	0.245	0.862
	**N**	35	35	35	33	35	34
$f \times D_p$	**R**	0.040	-0.247	0.037	-0.020	-0.158	-0.143
	**P-value**	0.82	0.153	0.833	0.912	0.365	0.42
	**N**	35	35	35	33	35	34

r: correlation coefficient. N: sample size. ADC: apparent diffusion coefficient. D_t: tissue diffusion. D_p: pseudo-diffusion coefficient. f: perfused fraction. $f \times D_p$: microvascular blood flow. F_b: blood flow. PS: capillary permeability–surface area product. v_e: interstitial volume fraction. v_b: blood volume fraction. v_d: extracellular volume fraction.

* $r \geq 0.4$ and $P<0.05$

** $r \geq 0.4$ and $P<0.001$

Table A3. Correlation between averaged DWI and DCE-MRI parameters from three MRI visits (Hot-spot region)

**Parameter**		Tumour T_1	F_b	PS	v_e	v_b	v_d
ADC	**R**	**0.520***	0.145	0.126	0.137	-0.146	0.203
	**P-value**	**0.001**	0.399	0.471	0.433	0.403	0.235
	**N**	**36**	36	35	35	35	36
D_t	**R**	**0.460***	0.042	0.056	0.194	-0.098	0.277
	**P-value**	**0.005**	0.808	0.749	0.264	0.575	0.102
	**N**	**36**	36	35	35	35	36
D_p	**R**	-0.039	0.070	0.138	0.081	-0.123	-0.172
	**P-value**	0.824	0.689	0.436	0.649	0.488	0.323
	**N**	35	35	34	34	34	35
f	**R**	0.343	0.329	0.368	0.103	-0.172	0.004
	**P-value**	0.044	0.054	0.032	0.562	0.331	0.982
	**N**	35	35	34	34	34	35
$f \times D_p$	**R**	0.179	0.251	0.258	0.158	-0.148	-0.053
	**P-value**	0.304	0.146	0.141	0.372	0.404	0.762
	**N**	35	35	34	34	34	35

r: correlation coefficient. N: sample size. ADC: apparent diffusion coefficient. D_t: tissue diffusion. D_p: pseudo-diffusion coefficient. f: perfused fraction. f×Dp: microvascular blood flow. F_b: blood flow. PS: capillary permeability–surface area product. v_e: interstitial volume fraction. v_b: blood volume fraction. v_d: extracellular volume fraction.

* $r \geq 0.4$ and $P<0.05$

Table A4. Repeated measures correlations between DWI and DCE-MRI parameters estimated from Cold-spot region.

**Parameter**		Tumour T_1	F_b	PS	v_e	v_b	v_d
ADC	$\mathbf{r_{rm}}$	0.103	-0.095	-0.026	0.126	0.263	**0.501****
	**df**	54	54	44	32	44	**42**
	**P-value**	0.449	0.484	0.863	0.477	0.077	**<0.001**
	**95% CI**	-0.279, 0.389	-0.349, 0.113	-0.335, 0.293	-0.161, 0.499	-0.035, 0.478	**0.217, 0.773**
D_t	$\mathbf{r_{rm}}$	0.03	0.029	0.043	0.113	0.092	0.266
	**df**	54	54	44	32	44	42
	**P-value**	0.826	0.829	0.778	0.525	0.545	0.081
	**95% CI**	-0.361, 0.284	-0.189, 0.264	-0.21, 0.309	-0.234, 0.61	-0.188, 0.303	0.019, 0.6
D_p	$\mathbf{r_{rm}}$	0.036	0.085	0.071	-0.047	0.029	-0.003
	**df**	47	47	37	26	37	35
	**P-value**	0.807	0.56	0.666	0.812	0.861	0.988
	**95% CI**	-0.125, 0.211	-0.085, 0.32	-0.352, 0.394	-0.501, 0.219	-0.383, 0.643	-0.202, 0.253
f	$\mathbf{r_{rm}}$	-0.031	-0.034	-0.082	0.109	0.102	0.116
	**df**	47	47	37	26	37	35
	**P-value**	0.834	0.814	0.618	0.582	0.536	0.493
	**95% CI**	-0.408, 0.277	-0.252, 0.217	-0.47, 0.275	-0.292, 0.442	-0.283, 0.431	-0.181, 0.372
$f \times D_p$	$\mathbf{r_{rm}}$	0.068	0.076	0.078	-0.023	0.002	0.039
	**df**	47	47	37	26	37	35
	**P-value**	0.64	0.602	0.638	0.909	0.99	0.818
	**95% CI**	-0.102, 0.251	-0.086, 0.291	-0.271, 0.325	-0.411, 0.228	-0.284, 0.497	-0.164, 0.295

r_{rm}: repeated measures correlation coefficient. df: degrees of freedom. CI: confidence interval. ADC: apparent diffusion coefficient. D_t: tissue diffusion. D_p: pseudo-diffusion coefficient. f: perfused fraction. f×Dp: microvascular blood flow. F_b: blood flow. PS: capillary permeability–surface area product. v_e: interstitial volume fraction. v_b: blood volume fraction. v_d: extracellular volume fraction.
** $r_{rm} \geq 0.4$, P<0.001, and bootstrapped 95% CIs excluded zero

Table A5. Repeated measures correlations between DWI and DCE-MRI parameters estimated from Hot-spot region.

Parameter		Tumour T_1	F_b	PS	v_e	v_b	v_d
ADC	**r_{rm}**	0.247	-0.012	0.004	0.36	0.078	0.34
	**df**	54	54	45	36	45	45
	**P-value**	0.067	0.929	0.979	0.026	0.603	0.019
	**95% CI**	-0.05, 0.511	-0.326, 0.248	-0.336, 0.364	-0.024, 0.622	-0.315, 0.425	0.153, 0.524
D_t	**r_{rm}**	0.246	0.022	0.063	0.254	-0.078	0.287
	**df**	54	54	45	36	45	45
	**P-value**	0.067	0.87	0.672	0.123	0.604	0.051
	**95% CI**	-0.012, 0.499	-0.279, 0.271	-0.191, 0.282	0.005, 0.476	-0.307, 0.181	0.09, 0.548
D_p	**r_{rm}**	0.048	-0.028	-0.085	-0.257	0.087	0.013
	**df**	51	51	43	34	43	42
	**P-value**	0.733	0.84	0.579	0.13	0.569	0.932
	**95% CI**	-0.25, 0.35	-0.34, 0.234	-0.44, 0.125	-0.736, -0.001	-0.4, 0.485	-0.565, 0.388
f	**r_{rm}**	-0.127	-0.147	-0.178	0.04	0.268	0.013
	**df**	51	51	43	34	43	42
	**P-value**	0.364	0.292	0.242	0.816	0.076	0.932
	**95% CI**	-0.388, 0.21	-0.461, 0.221	-0.526, 0.269	-0.567, 0.53	-0.208, 0.655	-0.279, 0.308
$f \times D_p$	**r_{rm}**	-0.002	-0.066	-0.083	-0.094	0.148	0.109
	**df**	51	51	43	34	43	42
	**P-value**	0.99	0.637	0.586	0.587	0.331	0.481
	**95% CI**	-0.352, 0.362	-0.438, 0.279	-0.329, 0.127	-0.566, 0.094	-0.255, 0.424	-0.435, 0.441

r_{rm}: repeated measures correlation coefficient. df: degrees of freedom. CI: confidence interval. ADC: apparent diffusion coefficient. D_t: tissue diffusion. D_p: pseudo-diffusion coefficient. f: perfused fraction. f×Dp: microvascular blood flow. F_b: blood flow. PS: capillary permeability–surface area product. v_e: interstitial volume fraction. v_b: blood volume fraction. v_d: extracellular volume fraction.

3 Supplementary Figures

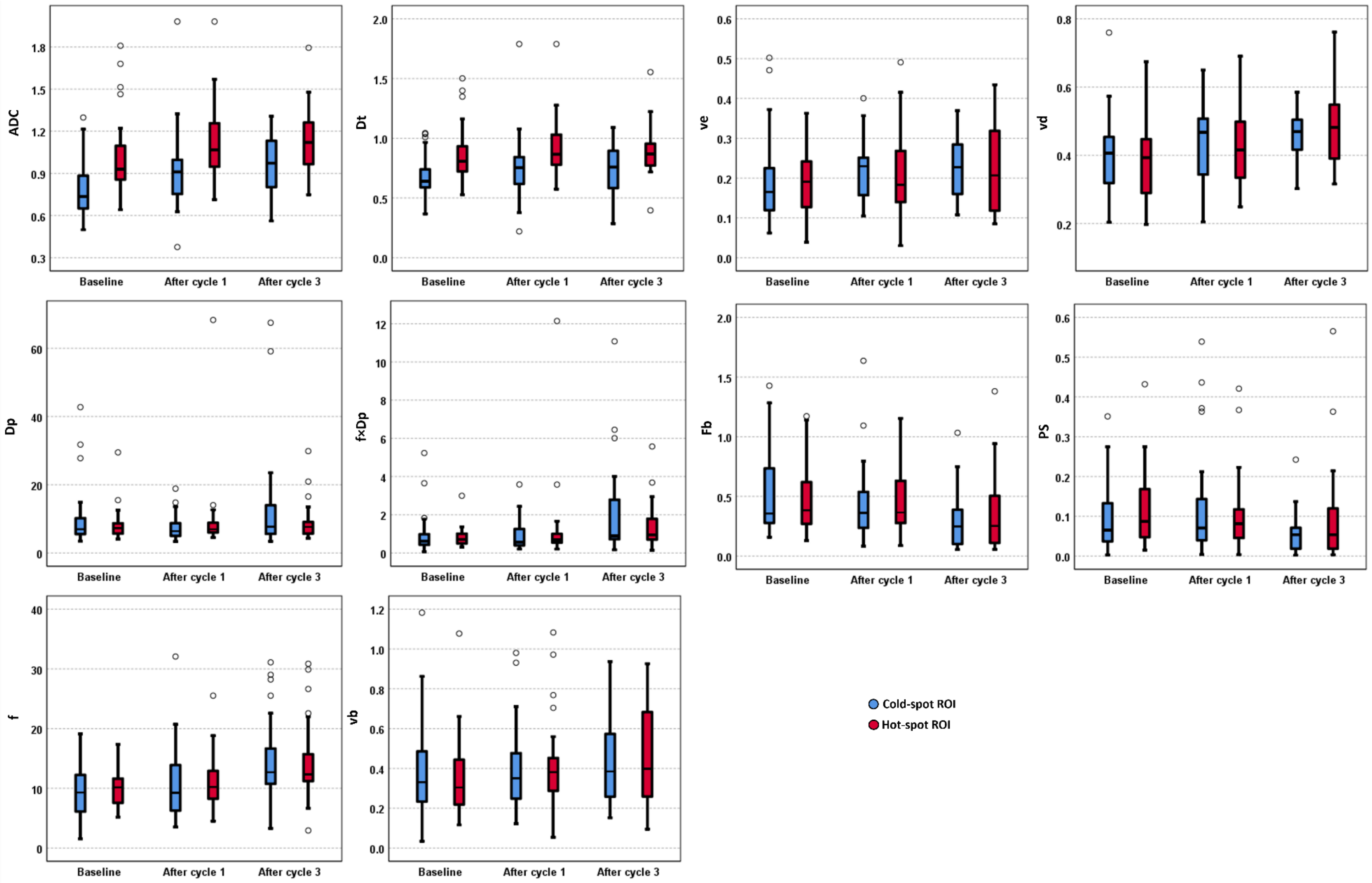

Figure A1. Evolution of DWI (ADC, D_t, D_p, f, and f×D_p) and DCE-MRI (F_b, v_b, PS, v_e, and v_d) parameters across the three MRI visits (Baseline, and after one and three cycles of NACT). Box plots illustrate the median and interquartile range values of all patients for cold-spot and hot-spot regions at each MRI visit. ADC: apparent diffusion coefficient. D_t: tissue diffusion. v_e: interstitial volume fraction. v_d: extracellular volume fraction. D_p: pseudo-diffusion coefficient. f×D_p: microvascular blood flow. F_b: blood flow. PS: capillary permeability–surface area product. f: perfused fraction. v_b: blood volume fraction.

References:

1. Partridge SC, Zhang Z, Newitt DC, Gibbs JE, Chenevert TL, Rosen MA, et al. Diffusion-Weighted Mri Findings Predict Pathologic Response in Neoadjuvant Treatment of Breast Cancer: The Acrin 6698 Multicenter Trial. *Radiology* (2018) 289(3):618-27.
2. Suo S, Lin N, Wang H, Zhang L, Wang R, Zhang S, et al. Intravoxel Incoherent Motion Diffusion-Weighted Mr Imaging of Breast Cancer at 3.0 Tesla: Comparison of Different Curve-Fitting Methods. *J Magn Reson Imaging* (2015) 42(2):362-70.
3. Almutlaq ZM, Wilson DJ, Bacon SE, Sharma N, Stephens S, Dondo T, et al. Evaluation of Monoexponential, Stretched-Exponential and Intravoxel Incoherent Motion Mri Diffusion Models in Early Response Monitoring to Neoadjuvant Chemotherapy in Patients with Breast Cancer—a Preliminary Study. *J Magn Reson Imaging* (2022) 56(4):1079-88.
4. Park HJ, Sung YS, Lee SS, Lee Y, Cheong H, Kim YJ, et al. Intravoxel Incoherent Motion Diffusion-Weighted Mri of the Abdomen: The Effect of Fitting Algorithms on the Accuracy and Reliability of the Parameters. *J Magn Reson Imaging* (2017) 45(6):1637-47.
5. Shukla-Dave A, Obuchowski NA, Chenevert TL, Jambawalikar S, Schwartz LH, Malyarenko D, et al. Quantitative Imaging Biomarkers Alliance (Qiba) Recommendations for Improved Precision of Dwi and Dce-Mri Derived Biomarkers in Multicenter Oncology Trials. *J Magn Reson Imaging* (2019) 49(7):e101-e21.
6. Eisenhauer EA, Therasse P, Bogaerts J, Schwartz LH, Sargent D, Ford R, et al. New Response Evaluation Criteria in Solid Tumours: Revised Recist Guideline (Version 1.1). *Eur J Cancer* (2009) 45(2):228-47.
